# Obstacles and opportunities for reducing dwelling size to shrink the environmental footprint of housing: tenants’ residential preferences and housing choice

**DOI:** 10.1007/s10901-021-09884-3

**Published:** 2021-09-13

**Authors:** Claudine Karlen, Anna Pagani, Claudia R. Binder

**Affiliations:** Laboratory for Human-Environment Relations in Urban Systems (HERUS), Environmental Engineering Institute (IIE), School of Architecture, Civil and Environmental Engineering (ENAC), École Polytechnique Fédérale de Lausanne (EPFL), Lausanne, Switzerland

**Keywords:** Space consumption, Housing preferences, Residential mobility, Sustainability, Switzerland

## Abstract

The environmental footprint of housing is greatly influenced by the size of a dwelling. Housing size is the result of households’ dwelling selections; accordingly, it is critical to consider residential preferences and choices to inform efforts towards housing sustainability. This study aimed to understand tenants’ preferences for and choices of housing size as one amongst several dwelling characteristics and identify obstacles and opportunities for reducing size in the light of promoting sustainable housing. We employed logistic regression models to analyse a survey with 878 Swiss tenants, and our results identify preference for large dwellings as a major obstacle for reducing dwelling size among affluent tenants. Conversely, tenants with lower income might be forced to move to a smaller dwelling due to financial constraints or attribute higher importance to the financial benefit of lower rents. However, financial disincentives along with substantial non-monetary costs of moving, such as the disruption of local bonds and the difficulty of finding a satisfactory dwelling, can outweigh the benefits of moving to a smaller dwelling. To overcome such obstacles, we suggest offering incentives and other facilitating measures for downsizing moves as well as ensuring an adequate supply of smaller dwellings capable of providing high living quality. We highlight the potential of studying housing functions to conceptualize dwellings fulfilling these requirements.

## Introduction

Housing contributes substantially to the human environmental footprint on a global scale (GlobalABC, IEA and UN 2019). The consumption of land, energy, materials and water as well as the production of waste and emissions by the residential sector impose manifold impacts on the natural environment (Lavagna et al., 2018; Williams, 2007).

The size of dwellings is a key factor in determining the consumption of resources and energy in housing (Heeren & Hellweg, 2019; Huebner et al., 2015; Lavagna et al., 2018; Saner et al., 2013; Williams, 2007). Several studies suggest that the per capita environmental footprint of housing increases with rising per capita floor space (Clune et al., 2012; Ellsworth-Krebs, 2020; Huebner & Shipworth, 2017; Huebner et al., 2015; Lorek & Spangenberg, 2019). To sustain the additional floor area, more resources are required in the construction and use phases, which dominate the environmental impact of a building during its life cycle. In the construction phase, additional dwelling space leads to a higher demand for land, materials and energy, while during the use phase, more energy is consumed, namely for space heating (Heeren & Hellweg, 2019; Lavagna et al., 2018; Saner et al., 2013; Williams, 2007).

In Switzerland, as well as globally, a significant increase in per capita living area has been observed in the last decades, which has been associated with an increase in the size of dwellings and a growing number of one- or two-person households, which requires more separate dwelling units (Bradbury et al., 2014; Williams, 2007). The unrestricted growth in per capita space consumption—from 34m^2^ in 1980 to 46 m^2^ in 2019 (Delbiaggio et al., 2018; FSO, 2019a)—has partly undermined the efforts to reduce the substantial share of Swiss final energy use attributed to buildings (Infras et al., 2019; Prognos, 2019), and is likely to do even more so in the future.

Hence, there is a need for planning and policy instruments that target a relative reduction of energy consumption (i.e. increasing energy efficiency) as well as an absolute reduction of domestic consumption by restricting further growth in or even reducing per capita floor space. Concerning the latter, scholars have stressed that policy interventions should begin with the sociocultural dimension of housing space consumption (Dowling & Power, 2012; Ellsworth-Krebs, 2020; Ellsworth-Krebs et al., 2021); on the one hand, household practices and visions of an ideal home can determine the materiality (i.e. the size) of the chosen dwelling; on the other hand, the supply of dwellings on the market as well as policy and institutional regulations can influence households’ dwelling choices (Pagani et al., 2020). The interplay of these factors has been the subject of a vast body of residential mobility literature describing how households adjust their housing consumption to meet changing needs (Rossi, 1955). However, research on residential relocation processes has thus far hardly addressed questions in the context of environmental sustainability, in particular regarding households’ space consumption.

With this paper, we aim to gain an understanding of households’ preferences for and choices of dwelling size and thereby identify obstacles and opportunities for reducing the latter. Such insights are crucial for reconciling a reduction of the housing environmental footprint with households’ preferences and needs.

On the basis of a survey with Swiss tenants, we first seek to understand what has led households to move to smaller or larger dwellings in the past and secondly analyse tenants’ stated willingness to move to a smaller dwelling in response to a shrinking household size. Our analysis addresses the following research question and sub-questions:

*What determinants of households’ relocation decisions present opportunities or obstacles for reducing housing size? *

- *What determinants have led households to reduce or augment their dwelling size during the last relocation?*
- *What are the determinants of tenants’ willingness to move to a smaller dwelling if their household were to shrink in size?*

To answer these questions, we proceed as follows. In the next section, we review relevant concepts in previous residential mobility literature in order to establish a theoretical framework and formulate hypotheses for our study. In the third section, we describe the methods used to analyse the tenant survey, the results of which we present in the fourth section. Before concluding, we discuss the theoretical and practical implications of our results in the fifth section and present the limitations of our study along with potential future research toward the goal of improving housing sustainability.

## Theory and background

### Residential mobility

Residential mobility describes the process whereby a household reacts to shifting housing needs and preferences and adjusts its residential situation through relocation (Mulder & Hooimeijer, 1999; Rossi, 1955). This process is influenced by an interplay of micro- and macro-level factors (Mulder & Hooimeijer, 1999; van Ham, 2012).

The relocation process is initiated by a *trigger* that induces a household’s desire to move (Mulder, 1996; Mulder & Hooimeijer, 1999). Triggers can arise from the micro- or macro-context. The micro-context represents the household or the individual, whose life-course is constituted of sequences of life events within different domains—such as education, labour, leisure, family and housing—termed trajectories or careers. As the trajectories of different life domains and household members evolve in parallel to each other, an event in one trajectory can induce a change in a household’s situation (Clark & Lisowski, 2017; Clark & Onaka, 1983; Clark et al., 1984; Dieleman & Schouw, 1989; Kan, 1999), which in turn can result in a shift in housing needs and preferences in order to accommodate the new situation (Mulder & Hooimeijer, 1999; van Ham, 2012). Furthermore, triggers for relocation can arise from the macro-context, which represents the ‘external’ environment that cannot be influenced by the household, such as the housing market and institutional situation (Brown & Moore, 1970; Clark & Onaka, 1983; Mulder & Hooimeijer, 1999). Such triggers include the expiration of a rental contract or the availability of a specific offer on the market.

In order to adjust to an altered situation, the household considers moving to a new dwelling. It can also decide to improve the current dwelling situation by restructuring its environment (e.g. purchasing a car to reduce the distance to work; Brown & Moore, 1970; Dieleman, 2001); however, this is not always an option. If the household has developed a desire to move, it will evaluate available vacancies on the market according to its preferences and choose the dwelling that best satisfies them (Brown & Moore, 1970). Numerous scholars have investigated preferences for certain types of housing in terms of dwelling, neighbourhood and location characteristics (Dieleman, 2001; Molin et al., 1996; van Ham, 2012; Wong, 2002). In their recent exploration of residential mobility in the Swiss context, Pagani et al. (2021) and Pagani and Binder (2021) introduced the notion of housing *function* (e.g. ‘shelter’; c.f. Table 6 in the Appendix for all functions) as a mediator between residential preferences (e.g. dream of the homely home) and housing form (e.g. detached suburban house). Residential preferences are determined by households’ life-course trajectories or residential biography, whereas the housing form includes a bundle of characteristics, one of them being dwelling size.

Some scholars regard the chosen dwelling as revealing the household’s preferences for dwelling type and environment (i.e. *revealed* preferences or *current* housing functions). However, due to the high cost of moving and limited availability of dwellings on the market, or a lack of knowledge thereof, there can be a discrepancy between *revealed* and *stated* (i.e. *ideal* housing functions) preferences (de Groot et al., 2011a, 2011b; Hooimeijer & Oskamp, 1996; Mulder, 1996; Pagani et al. 2021; van Ham, 2012). In fact, whether a desire to move is translated into action and what dwelling will be chosen depends on enabling and inhibiting factors arising from the micro- and macro-contexts. The enabling aspects of the macro-context are termed *opportunities* and refer to the offers available on the housing market. Inhibiting factors are denoted *constraints* and can emerge through conditions such as the accessibility of a certain location or eligibility criteria for subsidized housing. The set of available dwellings for a household is further influenced by *resources* and *restrictions* of the micro-context, i.e. the characteristics of a household that derive from the state of its parallel life-course trajectories (Mulder, 1996; Mulder & Hooimeijer, 1999; van Ham, 2012). More specifically, previous studies have investigated the role of characteristics such as income (Clark & Lisowski, 2017; de Groot, et al., 2011a, 2011b; Lu, 1998; Wanner, 2017), employment (Kan, 1999; van Ham, 2012), and age (Clark & Lisowski, 2017; Clark & Onaka, 1983; de Groot et al., 2011a, 2011b; Fiori et al., 2019; Lu, 1998) in the formation and realization of moving intentions. Furthermore, the functions fulfilled by the dwelling at the time of the move have been found to influence a tenant’s propensity to move (Pagani et al. 2021).

Depending on a combination of such enabling and hindering factors, moving entails substantial monetary and non-monetary costs, which is why relocation is only considered when a sufficiently strong trigger is present and the expected improvement of the situation outweighs the costs (Mulder, 1996; Mulder & Hooimeijer, 1999). The household’s *level of residential satisfaction* has been found to critically influence the probability to form a wish to move. The higher the household’s residential satisfaction relative to its dissatisfaction threshold, the less likely the household is to develop a wish to move, and the greater the cost of moving, the higher the dissatisfaction threshold (Speare, 1974). In other words, whether or not a certain trigger effectively induces an intention to move depends on the household’s level of satisfaction (Pagani et al. 2021).

**Fig. 1:**
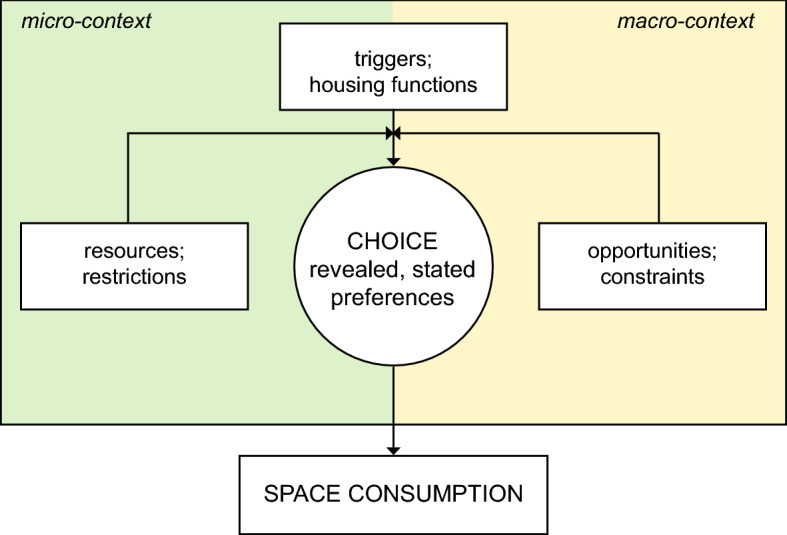
Theoretical framework employed in this study. The decision to move and the choice of a new dwelling are shown as a result of an interplay between triggers for moving and the ideal and current housing functions, resources and restrictions and opportunities and constraints. The latter arise from the micro- and from the macro-context, respectively, whereas triggers and housing functions are shaped by, both, the micro- and macro-context. The space consumption in housing constitutes the result of the residential choice

In this study, we adopt a model in which a combination of factors from the micro- and macro-contexts simultaneously determines a mobility outcome. Our model corresponds to the ‘risk approach’ elucidated in Mulder (1996), which is a ‘mainstream type of research’ (p. 216) to investigate determinants of the ‘risk’ to move using surveys that do not contain separate information on intentions to move and actual moves. Figure 1 depicts the theoretical framework used in this study. A trigger for moving deriving from the micro- or macro-context can represent a change in the housing function desired for the new dwelling (i.e. ideal function), which is itself simultaneously shaped by life-course trajectories and factors such as the housing market. In addition, enabling and hindering factors from the micro- and macro-contexts influence the translation of a moving intention into action and the choice of a new dwelling. Space consumption, which is associated with an environmental impact, is shown as the result of the decision to move and the choice of dwelling. The framework only includes the relationships relevant for the present empirical study, with no claim to completeness.^1^

### The Swiss housing context

Despite its economic strength, Switzerland features a comparatively high share of tenants (60% in 2017; Werczberger, 1997; FSO, 2019a, 2019b, 2019c). A specific quality of the Swiss rental market is rent control, which restricts rental owners’ ability to raise the rent in existing tenure contracts at will (Bourassa et al., 2010; Sager, 2018; Werczberger, 1997). Although this legislation protects tenants from excessive rents, it has led to substantial differences between existing rents and those negotiated in new contracts (i.e. ‘rent-gap’), the consequences of which include reduced residential mobility and a higher probability to live in a too large or too small dwelling (Sager, 2018). Another feature of the Swiss housing market is the low vacancy rates (Bourassa et al., 2010), which amounted to 1.72% across the country in 2020, with values below one per cent in urban cantons (Zürich, Genève, Basel-Stadt; FSO, 2019a, 2019b, 2019c). Although the vacancy rate has been rising for more than ten years (FSO, 2019a), the Swiss housing market is still characterized by a shortage of supply, in particular for affordable housing (Balmer & Gerber, 2018; Tranda-Pittion, 2009). One instrument counteracting this issue is housing cooperatives, which are partly supported by the state (Balmer & Gerber, 2018). Housing cooperatives aim to withdraw real estate objects from the speculative market and offer dwellings for cost rent. Furthermore, they often follow a social purpose, promoting participation, neighbourhood relations and social mixing among inhabitants. The admission of inhabitants is regulated to a variable extent in different cooperatives.^2^ Furthermore, certain cooperatives establish occupancy rules that oblige tenants to relocate when occupancy decreases below a certain threshold. In such cases, tenants are commonly given the opportunity to move to a smaller dwelling within the cooperative.^3^ The national market share of housing cooperatives is 8.4%; the share is higher in urban regions—including 16% in the canton of Zurich (FSO, 2019a).

Per capita dwelling space in Switzerland has substantially increased in the past few decades to reach an overall average of 46 m^2^ per person in 2019 (41m^2^ for renters and of 53m^2^ for homeowners; FSO, 2019a). Potential reasons for the growth in per capita dwelling space are, firstly, that households do not reduce their dwelling size when their size decreases, as empirical studies have shown (NZZ & Wüest Partner AG, 2018, 2019; Rey, 2015). The failure to downsize in case of reduced space requirements can have structural reasons, but also may be the result of a generalized preference for large dwellings (Clark et al., 1984; Delbiaggio et al., 2018; NZZ & Wüest Partner AG, 2018), which constitutes a second reason for high per capita space consumption in Switzerland. Thirdly, the number of one- or two-person households has been growing in the past several decades; whereas 12% of the population was living in one-person households in 1980, the share in 2018 had increased to 16% of the population, corresponding to 36% of all households. This proportion is projected to continue to rise in the future (FSO, 2019b). An increased number of small households leads to a higher demand for separate dwelling units and less sharing of space among household members. Therefore, the dwelling area per person in Switzerland is on average larger in one-person households than in households with two or more people (FSO, 2019a).

### Hypotheses

Based on our review of previous literature, we lay down a set of hypotheses for the tenant survey analysis. In line with the two research sub-questions, the first two hypotheses address the housing choice made with the past move (i.e. revealed preferences of housing size), and the remaining two hypotheses concern the willingness to move in response to a shrinking household (i.e. stated preferences).

#### H1

There is an overall trend of moving to larger dwellings, regardless of the change in household size.

#### H2

Whether dwelling size was augmented or reduced can be explained by a combination of the trigger event and changes in household size and housing functions.

#### H3

A minority of the tenants would be willing to move if their household size decreased.

#### H4

The willingness to move can be explained by the simultaneous effects of current housing functions, households’ sociodemographic characteristics, current dwelling size and dwelling owner and residential satisfaction.

## Methods

### Data collection

The data used in this study was obtained from a quantitative survey with tenants in Switzerland. This survey is part of the research project ‘Shrinking Housing’s Environmental Footprint (SHEF)’, supported by the Swiss National Science Foundation (SNSF) within the framework of the National Research Programme ‘Sustainable Economy: resource-friendly, future-oriented, innovative’ (NRP 73) under Grant [number 407340_172435]. Three real estate owners, namely Allgemeine Baugenossenschaft Zürich (ABZ), Société Coopérative d’Habitation Lausanne (SCHL) and Schweizer Mobiliar Asset Management AG (SM) are partners in the research. The survey was approved by the HREC (Human Research Ethics Committee) of EPFL and carried out by the LINK institute for market and social research in Switzerland, which selected a random sample of 3020 tenants of the three project partners covering the German and French language regions. The survey was conducted from September to November 2019 and was online based with a limited amount of CATI (Computer Assisted Telephone Interview) available for the elderly or people lacking internet access. The response rate was 32% for a total sample of 968 responses. The data were cleaned by inspecting cases and variables. Regarding the former, cases were deleted when answers to nominal and ordinal variables had a standard deviation of 0 across a block, e.g. respondents always checked the first answer option (straightliners), which resulted in deletion of 90 cases. To clean variables, we focused on the variables capturing current and previous household and dwelling size. Whilst data on the current dwelling size had been provided by the dwelling owners and associated to the ID of each survey participant prior to anonymization, the current household size was provided by the survey respondents and three outliers were set as missing, where consistency with other variables was not given. As for the previous dwelling, data on both, the dwelling and household size, were provided by the respondents, we proceeded as follows to detect outliers: we calculated the dwelling area per capita (m^2^/cap), its third quartile (Q_3_) and the interquartile range (IQR). We coded the variables previous dwelling size, previous household size as well as previous dwelling area per capita as missing in case the following condition was true: ${m}^{2}/cap > {Q}_{3}+3*IQR$. This led to 14 missing cases. Data cleaning resulted in a final sample of 878 cases. All treatment and analysis of the data was conducted with the software IBM SPSS Statistics for Windows, V26 (IBM Corp., Armonk, N.Y., USA).

### Structure, content and measures of the study

Our analysis of the tenant survey proceeded in two steps (c.f. Table 1). Firstly, we analysed the housing choices of the tenants’ past moves. We considered change in dwelling size as the variable of interest and related it to a set of independent variables.

**Table 1:** Structure of the study, including the analysed dependent and independent variables and the employed methods. The independent variables are classified according to their provenance from the micro- or macro-context of the residential mobility process (c.f. Fig. 1).

Section	Dependent variable	Independent variables	Micro- / macro-context	Method
Previous move	Change in dwelling size	Change in HH size	Micro	Binary logistic regression
		Change in housing functions	Micro/macro	
		Triggers	Micro/macro	
Reducing dwelling size when the HH shrinks	Willingness to move	Reasons for unwillingness	Micro/macro	Descriptive
		Current housing functions	Micro/macro	Multinomial logistic regression
		Household characteristics	Micro	
		Current dwelling size	Micro	
		Level of satisfaction	Micro	
		Dwelling owner	macro	

HH = household

Secondly, we considered the tenants’ stated residential preference by assessing their willingness to move to a smaller dwelling if their household were to shrink and their reasons for not being willing to move. We aimed to explain willingness to move with reference to a set of independent variables.

#### Variables

Three categories of information from the survey were used as variables in this study.

1. ***Housing functions*** of the previous and current dwellings (c.f. Table 6 in the Appendix). Respondents were asked to evaluate the importance of each function according to their description (and not the label) on a 5-point Likert scale. Housing functions were considered as interval variables in the statistical analysis.
2. ***Household and dwelling characteristics***, which included
  the sociodemographic characteristics of the household at the time of the survey and, for household size, at the time before moving (nominal variables, except for household size (interval variable))^4^;
  the size of the current and previous dwelling (interval variable);
  the dwelling owner (nominal variable); and
  the level of residential satisfaction with the current dwelling (ordinal variable evaluated on a 5-point Likert scale).
3. ***Housing choices***, captured with the
  trigger motivating the past relocation (nominal variable: a list of 20 events; see Pagani et al. (2021);
  prospect of moving within the next five years (nominal variable: 1 = *yes, 2* = *maybe, 3* = *no*);
  willingness to move to a smaller dwelling in case of a shrinking household (only for households counting more than one person; ordinal variable evaluated on a 5-point Likert scale); and
  reasons for not being willing to move and reasons potentially preventing those who were in principle willing to move from actually moving (open answers; two possible each).

#### Data transformation

A transformation of the survey data was performed, and the following additional variables were computed for the analysis:

- change in household (HH) size: a categorical variable was computed (*1* = *HH size decreased, 2* = *HH size increased, 3* = *HH size did not change*)
- change in dwelling size: a binary variable was computed (*1* = *the household reduced dwelling size, 0* = *the household did not reduce (i.e. augment) dwelling size*)
- residential satisfaction: five levels were aggregated to three categories (1 = *satisfied, 2* = *neutral, 3* = *unsatisfied*)
- change in housing functions: a categorical variable for each function was computed (*1* = *increase* (in the importance of the function)*, 2* = *decrease, 3* = *no change*)
- prospect of moving within the next five years: a binary variable was computed (*1* = *yes/maybe*; *0* = *no*)
- willingness to move in case of a shrinking household: five categories were aggregated into three (1 = *not willing, 2* = *neutral, 3* = *willing*)
- reasons for not being willing to move and reasons potentially preventing those willing to move in principle from actually moving: open answers were grouped, recoded and evaluated as multiple response sets

### Statistical analysis

Descriptive analysis of the dataset included computing the frequency of each nominal variable category and calculating the mean and standard deviation for metric variables.

To assess bivariate relations between nominal variables, we used the Pearson chi^2^ test. In cases of degrees of freedom (*df*) equal to one, we applied the Yates correction for a more conservative test statistic (Backhaus et al., 2018). To analyse relations between metric and nominal variables, we employed the Kruskal–Wallis test. We chose this non-parametric test because the metric variables did not follow a normal distribution.

To evaluate the combined effect of the independent variables on the dependent variables, we employed multiple regression models (c.f. Table 1 for the structure of the analysis).

Firstly, we conducted a binary logistic regression to explain the dichotomous dependent variable ‘the household reduced dwelling size with the previous relocation’. The independent variables used in the analysis comprised the changes in housing functions that occurred with the relocation as well as the trigger inducing the move. Since the relation between the trigger to move and the change in household size upon the move was not consistent (e.g. moving in with the partner did not always result in an increase in the household size; c.f. Table 8 in the Appendix), we considered change in household size as a separate independent variable in addition to the triggers. We computed different models incorporating different combinations of the predictor variables: (1) change in household size and change in housing functions; (2) change in household size and the triggers; and (3) all three groups of predictors. The change in household size was used in every model, as we assumed it to have the strongest effect on changes in dwelling size. Housing functions and triggers were simultaneously used in the last model. Although a link between triggers and housing functions exists (Pagani et al., 2021; Pagani & Binder, 2021), the two variables contain different facets of information such that one cannot be used in place of the other (e.g. not all triggers lead to a change in housing function). To avoid overfitting, not all variables and categories were included in the model. To select which changes in housing functions to include, we used the SPSS ‘forward stepwise’ algorithm (based on the significance of the conditional statistic) for inclusion of variables. Furthermore, we did not include triggers for which the number of observations was smaller than 25.

Secondly, we conducted a multinomial logistic regression to explain the tenants’ ‘willingness to move in case their household shrunk’. An ordinal logistic regression could not be performed because the assumption of parallel lines was not met by the data.^5^ Independent variables covered current housing functions, household- and dwelling-related micro-context variables and dwelling owner as a macro-context variable. We computed four different models of increasing complexity by adding the different variable blocks one by one.

We verified the following prerequisites of the data for both analyses. For each category of independent variables, the number of observations was equal or higher than 25. Further, we checked multicollinearity between independent variables by looking at the variance inflation factors (VIF), using the test implemented for linear regression in SPSS. All the VIFs were below a value of 10, which ensured sufficiently small multicollinearity (Backhaus et al., 2018).

## Results

### Sample characteristics

A description of the sociodemographic characteristics of the sample as well as the affiliation of the tenants with the three different dwelling owners and their space consumption is presented in Table 7 in the Appendix. Approximately half of the sample was constituted by women (54%) and men (46%), respectively, which is representative of the Swiss average (FSO, 2019b). The age categories 34–49 years and 50–64 years have the strongest representation in the sample (33% and 29%, respectively), followed by the categories of 65 years and older (21%) and 33 years and younger (17%). The slight overrepresentation of middle-aged and old people compared to the Swiss population is coherent with the fact that only adult tenants were surveyed (FSO, 2019b). Half of the respondents were married or living in a couple and roughly a quarter each were single or separated, divorced or widowed. Less than a third (28%) of the households had children. Households with one (33%) or two persons (35%) were most common, followed by those with three to four persons (27%) and a minority of households with five or more members (5%), which also coincides with national statistics (FSO, 2019b). Most respondents held either a professional school (39%) or a university degree (40%). A third of the households earned an annual income below CHF 60 K, 30% between CHF 60 K and 88 K and 21% between CHF 88 K and 120 K. The higher income categories are less frequently represented, with 10% and 6% earning CHF 120 K–165 K and more than CHF 165 K, respectively. Since the income categories in the survey were chosen differently from those in national statistics (FSO, 2019c), the values cannot directly be compared, but lower income categories are likely to be represented slightly more frequently than in national statistics. This is likely due to the high percentage of tenants from cooperatives in the sample who tend to have a lower income than those in the private rental market (see e.g. Allgemeine Baugenossenschaft Zürich, 2019). The three dwelling owners were represented by approximately a third of the respondents each (33.5%, 39.5% and 27% of the tenants renting from ABZ, SCHL and SM, respectively). The mean per capita floor space in the sample amounts to 46m^2^. This value is equal to the Swiss average but higher than the average among Swiss renters of 41m^2^ per person (c.f. Sect. 2.2). This might be because the sample represents only three different dwelling owners.

### Revealed preferences: Past housing choice

#### Change in space consumption (H1)

Table 2 shows the change in space consumption and change in household size resulting from the households’ last relocation.

**Table 2:** Change in space consumption with the previous move and its relation to the change in household size.

Full sample			Change in m^2^			Change in m^2^/cap		
	n	%	% reduced	% augmented	Sign.	% reduced	% augmented	Sign.
	864 / 862	100.0	40.0	60.0		29.0	71.0	
by* change in HH size*								
decreased	337	39.1	60.2	39.8	***		90.2	***
increased	137	15.9	18.2	81.8	***		25.5	***
no change	388	45.0	30.2	69.8	***		69.8	–

***Indicates the 1% significance level

In line with H1, although only 16% of the households grew, more than half of the relocations resulted in an increase in dwelling space (unit size as well as space per person). The majority (82%) of the households that grew moved to larger dwellings, as did 70% of the households that did not change in size and nearly 40% of the households whose size decreased.

We also observe that although 60% of the households that shrunk reduced the size of their dwelling, most of them (90%) increased their per capita space consumption. In smaller households, rooms such as kitchens or living rooms are shared among fewer people, which is why per capita space consumption can increase even if the overall dwelling size decreases (Williams, 2007).

#### Predictors of change in space consumption (H2)

Having found that more than half of the previous relocations in our sample resulted in an increase in dwelling space, we hereafter investigate the combined effect of changes in household size, changes in housing functions and triggers on the dichotomous variable ‘the household reduced dwelling size’. Table 3 displays the odds ratios (OR) of the significant regression parameters in the binary logistic regression model, which are ranked by the strength of their effect.

**Table 3:** Ranked odds ratios of the significant parameters in the binary logistic regression model for predicting the likelihood of having reduced dwelling size with the last relocation

Variable	OR (sign.)	95 % confidence interval	
		Lower value	Upper value
Children leaving home	5.706***	2.149	15.156
Divorce, separation, loss of partner	4.274***	1.927	9.481
Need for autonomy	2.666*	0.983	7.225
Decrease in HH size	2.275***	1.578	3.280
Rent too high	1.886*	0.934	3.809
Status symbol -	1.849***	1.185	2.886
New child	0.104***	0.040	0.269
Lack of space	0.122***	0.040	0.373
Opportunity to rent	0.395***	0.210	0.744
Status symbol +	0.623**	0.399	0.973
Privacy +	0.658**	0.445	0.973

***, ** and * indicate the 1%, 5% and 10% significance levels, respectively;

HH = household;

The plus or minus sign after a housing function indicates the increase or decrease, respectively, of the importance of that function for the household with the past relocation.

The results firstly show that triggers associated with a change in household size are key in explaining changes in dwelling size. The strongest effect on the dependent variable is exerted by the ‘birth of a child’, which implies the growth of the household and significantly reduces the likelihood of moving to a smaller dwelling (OR = 0.10). Triggers related to a shrinking household size—i.e. ‘children leaving home’, ‘divorce, separation or loss of partner’ and ‘need for autonomy’—significantly augment the probability of reducing dwelling size by factors 6, 4 and 3, respectively. Accordingly, a ‘decrease in household size’ with the past move also significantly increases the probability of moving to a smaller dwelling (OR = 2.3).

Secondly, we observe that a strong effect applies to ‘lack of space’, which diminishes the probability of a reduction of dwelling size without necessarily implying a growth in household size (c.f. Table 8 in the Appendix). Two additional triggers not related to a change in household size have a slightly weaker effect on the dependent variable; tenants moving for an ‘opportunity to rent’ were less likely to reduce the size of their dwelling (OR = 0.4), whereas a ‘too high rent’ shows the opposite effect (OR = 1.9).

Finally, the functions ‘status symbol’ and ‘privacy’ both exhibit a significant influence on the dependent variable. A decrease in the importance of a place for ‘exhibiting’ (i.e. ‘status symbol’) increases the likelihood of reducing dwelling size (OR = 1.9), and the opposite holds for an increase in the importance of this function (OR = 0.6). The same effect applies for a place fulfilling the ‘family’s needs’ (i.e. ‘privacy’; OR = 0.7).

In addition, Table 11 in the Appendix compares the described model (i.e. model 3) with two models using subsets of the independent variables: the change in household size and change in housing functions (model 1) and the change in household size and triggers (model 2). Model 3, which includes all three blocks of independent variables, shows the highest explanatory power (Nagelkerke pseudo R^2^ = 0.38) and the lowest AIC (Akaike Information Criterion) value, which implies that all the included independent variables add to the explanatory power of the model without the latter being offset by the increasing complexity of the model.

### Stated preferences: Reducing dwelling size when the household shrinks

#### Willingness to move (H3)

Having looked at the tendency to move to smaller or larger dwellings with the past move, in this section, we verify the hypothesis that the sample would exhibit a small propensity to move due to a shrinking household (H3). Table 4 shows the percentage of tenants in the sample that would be ‘not willing’, ‘neutral’ and ‘willing’ to move. In line with H3, 25% of the respondents were willing to move in case their household shrunk, 36% were undecided and 39% were not willing to move.

**Table 4:** Frequencies of categories of willingness to move to a smaller dwelling in response to a shrinking household

	Total	Not willing	Neutral	Willing
n	570	220	206	143
%	100	38.6	36.3	25.1

**Fig. 2:**
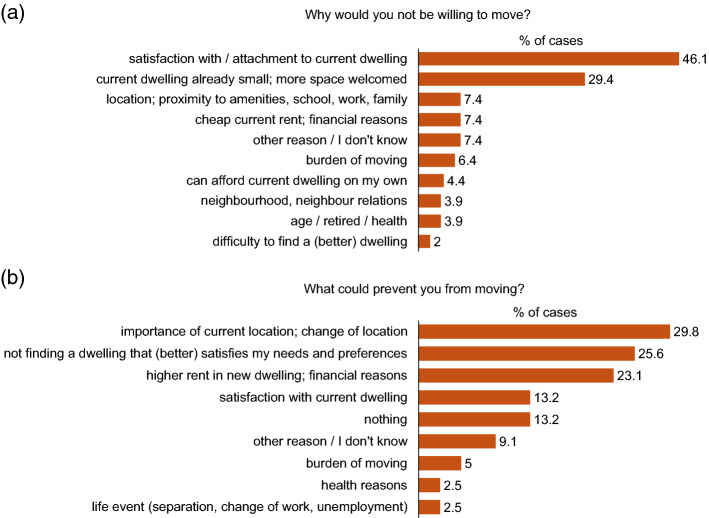
Multiple response frequencies of text answers to the questions a) Why would you not be willing to move in case your HH size decreased? (n = 204) and b) In case you were willing, what could prevent you from moving? (n = 121)

Figure 2 presents the various answer categories to the questions ‘Why would you not be willing to move in case your HH shrunk?’ and ‘If you were willing, what could prevent you from moving?’ derived from the text answers. The predominant reason for not being willing to move was satisfaction with the current dwelling situation, including location and neighbourhood, which together were mentioned by 57.4% of the respondents. In other words, the perceived necessity to move in case of excessive dwelling space is small, such that giving up a satisfactory housing situation is not worthwhile (H3). Furthermore, the liberation of space in case of a shrinking household is not necessarily perceived as a deterioration of the housing situation, i.e. the respondent would not prefer less space. Rather, a decrease in household size can lead from a suboptimal condition to a more desirable state in case more space is preferred. This is evident from the tenants who stated that their current dwelling was already small and/or they would welcome more available space, which represents the second most frequent reason for not being willing to move (29.4% of the respondents). In this case, no necessity to move is perceived at all. Satisfaction with the current dwelling situation is also among the more frequent reasons that could prevent tenants who are in principle willing to move from actually moving. Preponderant here is the importance of dwelling location, such that a change in location could prevent tenants from moving.

The financial aspects of relocation appeared to be important in the decision to move. Moving to a smaller dwelling intuitively implies a reduction in rent. Nevertheless, 7.4% of the respondents mentioned an inexpensive current rent (i.e. financial limitations) as a reason for not being willing to move, and 23.1% indicated a higher rent in the new dwelling as a reason potentially preventing them from moving. On the other hand, a small fraction (4.4%) of the respondents explicitly stated that they could afford their dwelling on their own if the household were to be reduced; in other words, for this subsample, reduction of the rent would not be an incentive to move.

The difficulty of finding a suitable new dwelling was mentioned by only 2% of the respondents as a reason for not being willing to move (least frequent reason) but is the second most frequently cited reason potentially preventing tenants who are in principle willing to move from actually moving (25.6%). Furthermore, the burden of the moving process was named with similar frequencies in both questions (6.4% and 5%), standing among the less frequently mentioned reasons. Also potentially referring to the burden of moving, some tenants stated their age, retirement or health as a factor (potentially) preventing them from moving (3.9% and 2.5%, respectively). The last category of reasons potentially preventing tenants from moving is life events (e.g. the loss of employment), which was mentioned by 2.5% of the respondents.

#### Predictors of the willingness to move (H4)

Knowing that only a quarter of the respondents would be willing to move if their household were to shrink, we hereafter explain the willingness to move with current housing functions, household and dwelling-related micro-context variables and the dwelling owner as a macro-context variable (c.f. Table 1). Table 5 presents the odds ratios of the significant parameters in the multinomial logistic regression model ranked by the strength of their effect on the probability of being willing to move (i.e. ‘willing’ against ‘not willing to move’). Willingness to move is most strongly predicted by the household income and the dwelling owner. Compared with those in the second lowest income category (60 K – 88 K CHF/y), tenants in higher income categories were less likely to be willing to move, with the inhibiting effect increasing in correlation with income (odds ratios between 0.5 and 0.1). The bivariate analysis shows a congruent result (c.f. Table 12 in the Appendix). Concerning the dwelling owner, the probability of ABZ tenants being willing to move was approximately a third as high as that of SCHL tenants, and SM tenants were approximately half as likely to be willing to move as ABZ tenants. The bivariate analysis reveals the same tendency (c.f. Table 12 in the Appendix). Lastly, several housing functions show a significant relation with not being willing to move. More specifically, a higher importance of the functions ‘status symbol’ and ‘permanence’ led to a lower probability of the tenant to be willing to move in case of a shrinking household (OR = 0.6 and OR = 0.7, respectively). However, we also observe that a higher importance of the functions ‘production-consumption’ and ‘self-representation’ increased the likelihood of being willing to move (OR = 1.9 and OR = 1.5, respectively).

**Table 5:** Ranked odds ratios of the significant parameters of the multinomial regression for predicting the category ‘willing to move’ with reference to the category ‘not willing to move’

Variable	OR (sign.)	95% confidence interval	
		Lower value	Upper value
Prospect of moving within the coming 5 years	2.068**	1.125	3.800
Production-consumption	1.858***	1.184	2.916
Self-representation	1.456**	1.055	2.009
Area of dwelling [m2]	1.025***	1.008	1.042
Annual income above 165K CHF	0.095***	0.027	0.337
Owner SCHL	0.296***	0.150	0.582
Annual income 120K – 165K CHF	0.331**	0.134	0.818
Owner SM	0.414**	0.191	0.895
Annual income 88K – 120K CHF	0.534*	0.257	1.108
Permanence	0.555***	0.402	0.767
Status symbol	0.710**	0.526	0.958

***, ** and * indicate the 1%, 5% and 10% significance levels, respectively

The ‘prospect of moving within the next five years’ shows the strongest enhancing effect on the probability of being willing to move. Tenants foreseeing a move were approximately twice as likely to be willing to move than those who did not expect a relocation. Even if a move is planned for another reason than a shrinking household, the idea of relocating might already be more familiar for these tenants, resulting in a higher willingness to move in case of a shrinking household. Finally, a larger area of the current dwelling positively influences the willingness to move, with an odds ratio close to 1 (OR = 1.03).^6^

Contrary to previous studies, the tenants’ age did not emerge as a significant predictor of the category ‘willing to move’. Age only shows a significant effect between the categories ‘not willing’ and ‘neutral’ (c.f. Table 13 in the Appendix), thereby indicating that even if they were not clearly willing to move, tenants aged 50–64 years seemed to show a less strong aversion to the idea of relocation than tenants aged 34–49, which may be because the former age group constitutes the period when children leave the parental home and parents might newly orient themselves. The presence of children only shows a significant effect on the category ‘neutral’. Households without children were more likely to be ‘neutral’ than ‘not willing’ compared with those with children (c.f. Table 13 in the Appendix). Since children can present an additional burden to moving (Mulder & Hooimeijer, 1999), their absence appears to reduce the aversion to moving but does not significantly enhance the willingness to move. Marital status appears to have no significant effect on the willingness to move. Even though satisfaction with the current dwelling was cited as an important reason for not being willing to move (c.f. Sect. 4.3.1), the level of satisfaction with the current dwelling does not appear as a significant predictor of the willingness to move in either the bivariate or multivariate analysis. It must also be noted that 80% of the respondents were rather or absolutely satisfied with their current dwelling (c.f. Table 12 in the Appendix).

Compared with the three additional models computed with subsets of the independent variables, the full model exhibits the highest explanatory power (Nagelkerke pseudo R^2^ of 0.27) and the lowest AIC (c.f. Table 13 in the “Appendix”). Furthermore, the significance and strength of the predictors in the chosen model (‘not willing’ vs. ‘willing’) display more significant and stronger effects than for the model including the category ('not willing’ vs. ‘neutral’).

## Discussion

Based on the premise that a reduction of housing size contributes to diminishing the environmental footprint of housing, the goal of this paper is to understand tenants’ preferences and choices regarding housing size and to identify obstacles and opportunities to reduce space consumption. In this section, we first discuss the findings from the tenant survey showing how different factors influence the decision to move and the choice of dwelling size. Secondly, we synthesize obstacles and opportunities for a reduction of housing size in the context of the Swiss rental market before we acknowledge the limitations of our analysis and illustrate possible paths for future research.

### Exploring tenants’ preferences of housing size

#### The choice of housing size in perspective

In the first part of the analysis, we investigated tenants’ revealed preferences for housing size. We found that more than half of the reported relocations resulted in an increase in dwelling space, which is in accordance with findings in previous studies (e.g. Clark et al., 1984) and validates our first hypothesis (H1).

The fact that a substantial proportion (40%) of the households that had decreased in size and the majority of those that had not changed size moved to larger dwellings implies a general preference for larger dwellings (c.f. Table 2). The likelihood of downsizing was related to several independent variables. Whereas triggers associated with a change in household size accordingly influenced the likelihood of reducing dwelling size (i.e. a shrinking household led to an increase of the probability to reduce space consumption and vice-versa), an opportunity to rent significantly decreased the probability of moving to a smaller dwelling, and the opposite was the case for relocations due to a too high rent (H2; c.f. Table 3). Moving for an opportunity presumably only happens when dwelling characteristics can be improved (Clark & Onaka, 1983), whereas moving due to excessively high rent implies the need to solve a problem whereby the household is financially limited in its choice of dwelling (Pagani et al., 2021). This corroborates the hypothesis of a preference for larger dwellings when an opportunity is available and implies that moving to a smaller dwelling is the result of a constraint.

Changes in two of the nine housing functions showed a significant effect on the odds of moving to a smaller dwelling (c.f. Table 3). This is in line with previous research indicating that housing functions determine the material behaviour of housing (i.e. housing characteristics such as size; (Pagani & Binder, 2021). More specifically, results indicate that an increase in the importance of a dwelling as a ‘credential for esteem’ or a place for ‘family’s needs’ lowers the likelihood of reducing its size. The first case reflects the ‘status symbol’ as a place for comfort, manifesting itself with features such as a growing amount of indoor facilities (e.g. library, exercise rooms; (Pagani & Binder, 2021) and with the potential to prove sophistication or classiness and respectability toward strangers (Dowling & Power, 2012). However, the second case is more controversial. A private place is defined as a place for the ‘family’s needs’ where ‘recreation preferably happens outside’ (c.f. Table 6 in the Appendix). On the one hand, saving space for leisure activities could suggest a reduction in housing size; on the other hand, an increase in the relevance of meeting a *family’s needs* might entail more spacious homes to satisfy the requirements of all family members and reconcile feelings of independence *and* familial togetherness within the home (e.g., one room per child; see Table 6 in (Pagani & Binder, 2021); Dowling & Power, 2012; Ellsworth-Krebs et al., 2021).

#### The willingness to move in perspective

In the second part of the analysis, we assessed tenants’ stated willingness to move to a smaller dwelling if the size of their household were to shrink and explored the determinants that influenced the latter.

We considered reduction in household size as an event in the household trajectory constituting a potential micro-context trigger for the formation of an intention to move. The survey results demonstrated that for almost 40% of the respondents, this trigger would not be sufficiently important to outweigh the expected cost of moving or was not regarded a trigger at all (H3; c.f. Table 4). This result is in accordance with the findings of a recent study on residential mobility in Switzerland in which only 5% of the respondents mentioned an excessively large dwelling as a reason for moving (NZZ / Wüest Partner AG, 2018). Nevertheless, our analysis showed some potential for reducing dwelling size, as 25% of the questioned tenants would be willing to move if their household were to shrink.

Based on our review of the literature, responses to the trigger event of a shrinking household were hypothesised to be determined by the function(s) fulfilled by the dwelling as well as enabling or hindering factors arising from the micro- and macro-context (H4).

In agreement with previous research, housing functions were found to influence the effectiveness of such a trigger in different ways (Pagani et al., 2021; c.f. Table 5). More specifically, the likelihood of being willing to downsize in response to a shrinking household was significantly higher for tenants attributing a stronger importance to ‘production-consumption’ and ‘self-representation’ of their dwelling but significantly lower for those giving higher values to ‘status symbol’ and ‘permanence’. The results are coherent with the definition of ‘production-consumption’ as a place for basic activities (i.e. eating, laundering), which require less space for a smaller household. For the function ‘status symbol’, the results agree with the interpretation given in the first part of our analysis and thereby indicate that the relevance of this function for a tenant has an influence both on its residential preferences and housing choice. Comparing the results to the findings of Pagani and colleagues (2021) can offer keys for interpretation of the effects of the other two functions. On the one hand, and in agreement with our findings, their research showed that tenants who attributed more importance to the function ‘self-representation’ had moved predominantly after a divorce or in response to excessively high rent, both of which imply a reduction in household size (see also previous section). On the other hand, and controversially, the authors indicate that the past moves of tenants who considered their dwelling a permanent place was triggered by a ‘dwelling too small’, a forced move (e.g., demolition), and most strongly a shrinking household (i.e. leaving the parents or having the children leaving the nest). These results highlight the relevance of a household’s *residential biography*, thereby indicating that tenants who had already adjusted their dwelling size in their previous move might be more reluctant to move again (and reduce their dwelling size).

Three main restrictions to downsizing moves were revealed in the micro-context. Firstly, we ascertained that satisfaction with and attachment to the current dwelling situation, including its location and neighbourhood, could prevent tenants from moving (c.f. Fig. 2). As elaborated in Mulder (1996) and Mulder and Hooimeijer (1999), the sentimental value of a dwelling and local bonds formed within the daily activity space can discourage households from moving. Secondly, we found increased household income to significantly lower the probability of being willing to move (c.f. Table 5). The strong influence of this predictor was not expected, as opposing qualitative effects of income have been suggested in the literature (de Groot et al., 2011a, 2011b; Lu, 1998; Wanner, 2017). Our results suggest that less affluent households may have less freedom to cope with a reduction of the number of household members who financially contribute to the rent and may be forced to move to a smaller—thus cheaper—dwelling, as was also suggested by Clark and Lisowski (2017). For tenants in higher categories of household income, the latter did not appear to be a factor promoting moves but rather for remaining in the current dwelling. Thirdly, retirement and old age were mentioned as reasons for not being willing to move or potentially preventing a move (c.f. Fig. 2). Obstacles for old people previously mentioned in literature include the rupture with a familiar environment, such as access to services and the social network, an uneasiness with change, and financial limitations (Delbiaggio et al., 2018; Neuhaus et al., 2016). Such findings illustrate the non-monetary cost of moving and are in line with other studies that found that the propensity to move was lower with higher age (Clark & Lisowski, 2017; de Groot et al., 2011a, 2011b; Lu, 1998). However, it must also be noted that age was not a significant predictor of the willingness to move in our regression model (c.f. Table 13 in the Appendix). Considering that tenants might have a higher propensity to move shortly after retirement than due to older age (Fiori et al., 2019), this finding could have resulted from an excessively broad definition of the older age category for the analysis (i.e. 64 years and older).

In the macro-context, a constraint for leaving a satisfactory dwelling in case of household reduction was the difficulty of finding a new dwelling with equal or better characteristics, which represents an additional important cost of moving (c.f. Fig. 2). Further, the preoccupation of having to pay a higher or equal rent in a smaller dwelling—a potential consequence of the Swiss rent control legislation—was found to constrain tenants from moving. In addition, the dwelling owner appeared to significantly influence the willingness to move in a manner representing both constraints and opportunities (c.f. Table 5). Occupancy rules for tenants benefiting from cost rent oblige them to move to a smaller dwelling when the household shrinks, whereas the absence of such rules favours remaining in the current dwelling, as might be the case for tenants of SCHL. The results also suggest that the practice of assisting tenants in finding a new dwelling within the cooperative positively influences the willingness to move, as reflected in the case of tenants of ABZ (c.f. Tables 5 and 13 in the Appendix).

### Obstacles and opportunities for reducing housing size

**Fig. 3:**
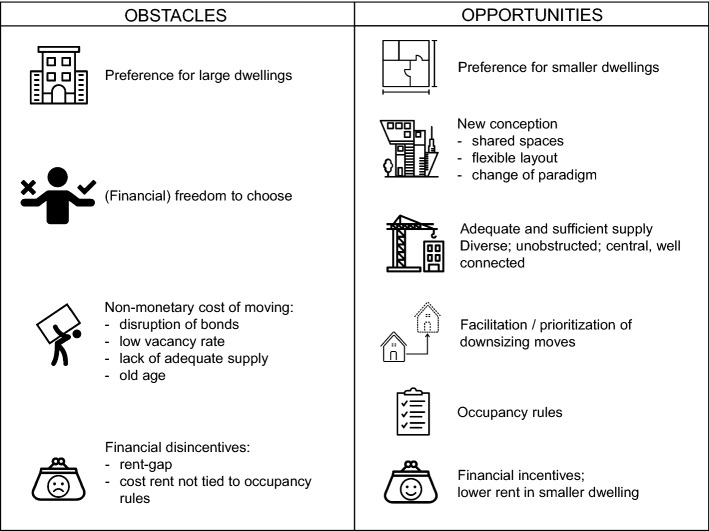
Overview of obstacles and opportunities for reducing housing size as synthesised from the results of the survey analysis

Based on our findings, we put forward several obstacles and opportunities for reducing housing size in the Swiss rental context to serve as inspiration for “invisible energy policies” (Royston et al., 2018), meaning policies that go beyond the sole enhancement of energy efficiency and aim at an absolute reduction of resource consumption by limiting housing space consumption. An overview of the identified aspects is presented in Fig. 3.

#### Reshaping preferences

A major obstacle for reducing housing size is the preference for large dwellings exhibited by a large proportion of respondents. In combination with sufficient financial resources and the freedom to choose one’s dwelling, such preference leads to a low propensity to move to a smaller dwelling. Tenants who tend to adhere to this logic have higher income and can be characterised, according to an additional analysis shown in Table 14 in the Appendix, as young or middle-aged, living as couples or married, and renting from the private market (i.e. SM) or living in a cooperative without occupancy rules (i.e. SCHL). For these tenants, we assume it would be difficult to present incentives (financial or other) to reduce their space consumption so long as reduced dwelling space is equated with a loss of dwelling quality and thus quality of living. Therefore, we articulate the need to overcome current housing standards and develop solutions that fulfil households’ preferences and needs while efficiently using space such that living in a smaller dwelling would no longer be the result of a constraint but rather a choice even for more affluent households.

The ascertained relationship between housing functions and stated or revealed dwelling size preferences corroborates the existence of a link between practices and values and resource use (Dowling & Power, 2012). Understanding which housing aspirations require more space to be satisfied can support the conception of dwellings fulfilling the same functions with a reduced consumption of space. Our findings identified the high importance of the functions ‘privacy’, ‘status symbol’ and ‘permanence’ to be an obstacle for reducing housing size, which could be tackled as follows:

- *Privacy*: To enable households to have separate rooms for separate uses while reducing their personal space, residential buildings could provide shared rooms and facilities (e.g. a workshop room or a music room; Huebner & Shipworth, 2017; Pattaroni & Marmy, 2016). Such rooms could still preserve households’ privacy, such as via a room-rental system. Furthermore, architectural solutions and sound-proofing could be employed to provide senses of privacy for individual family members (Dowling & Power, 2012; Ellsworth-Krebs, 2020; Ellsworth-Krebs et al., 2021).
- *Status symbol*: Shrinking the size of a ‘status symbol’ requires acting on the culture and society in which this function is rooted. Media, architects and designers can play a powerful role in forming expectations of an ideal home (Ellsworth-Krebs, 2020; Ellsworth-Krebs et al., 2019, 2021) and thereby shape a new ‘sustainable’ status symbol through the advertisement of dwellings of small size *and* a high quality of living.
- *Permanence*: Attachment to a dwelling and the neighbourhood play key roles in defining what housing is. A dwelling with a flexible layout capable of adapting to the evolution of the household could enable a reduction of space consumption while also relieving the burden of moving. In case of a shrinking household, excess space could be placed at the disposal of additional users (e.g. Beyeler, 2017; Ellsworth-Krebs, 2020), which might be especially beneficial for the elderly.

#### Mitigating the cost of moving

An opportunity for reducing housing size is evinced in the minority of tenants who would be willing to move if their households shrunk either because the financial incentive of paying less rent outweighs other preferences or because they see an advantage in having a smaller dwelling. In the first case, these are generally tenants with limited financial means living in a cooperative with occupancy rules (i.e. ABZ), thus having less freedom to choose their dwelling, such as older tenants and those living alone (c.f. Table 14 in the Appendix). In the second case, these are tenants who tend to change their dwelling in order to adapt to their current life-stage or aspirations (function ‘self-representation’) or those who regard their dwelling as a place that serves mostly for the basic needs of the household (function ‘production-consumption’). However, these tenants can be deterred from relocating due to the high monetary and non-monetary costs of moving; the elderly may be particularly affected by such costs.

An important non-monetary cost of moving in the micro-context is the disruption of household members’ sentimental attachment to the current dwelling and bonds formed within the daily activity space (c.f. Fig. 2). Arising from the macro-context, a second non-monetary cost is the difficulty of finding a suitable dwelling, which is likely a result of the low vacancy rates in Switzerland, especially in urban areas, but potentially is also due to an inadequate supply of small dwellings for the growing number of single- and two-person households of both young and elderly tenants (ETH Wohnforum—ETH CASE 2016; Neuhaus et al., 2016). The lack of enough and adequate supply of small dwellings has previously been mentioned as a barrier to downsizing for the cases of the UK and Germany (Huebner & Shipworth, 2017; Lorek & Spangenberg, 2019) and likely represents an obstacle for downsizing also in Switzerland.

To relieve the non-monetary costs of moving, a basic requirement would be an appropriate and sufficient supply of small dwellings that fulfil the needs of diverse life-designs and household sizes (i.e. singles, patchwork families, elderlies with special requirements, etc.). Furthermore, the aspiration across age groups for living centrally, well connected and in proximity to daily activity spaces and social networks stood out in this research and was also put forward in other articles (e.g. Neuhaus, Ruetz and Roth, 2016; Birrer & Glaser, 2017). In light of the already limited space in (urban) centres, this finding implies a need for denser, area sparing construction or the formation of new liveable centres with diverse utilization. To promote this, corresponding incentives and rules for investors could be established (for more details, see Institut für Wirtschaftsstudien Basel, 2016; Ellsworth-Krebs, 2020; Huebner & Shipworth, 2017), which would lead to a reduction of resource consumption and at the same time enable more people to live in desired areas. To minimize the disruption of local bonds, a mix of dwellings of different sizes would be needed in a building project, such that relocating to a smaller dwelling within the same complex would be possible in case of household shrinkage (Institut für Wirtschaftsstudien Basel, 2016). To further facilitate relocation, rules for prioritizing moves to smaller dwellings and/or a minimum occupancy are imaginable not only in housing cooperatives but also more widely. In addition, counselling for moves could be provided from an institutional side, which could be especially beneficial for the elderly (Institut für Wirtschaftsstudien Basel, 2016).

Finally, monetary costs of moving also present obstacles for reducing dwelling size. The rent-gap engendered by the rent control legislation in Switzerland (c.f. Sect. 2.2) presents a financial disincentive or even restriction for moving. A recent empirical study about the effect of the rent-gap on residential mobility in Switzerland did not find the former to be a significant predictor of living in a too-large dwelling (Sager et al., 2018); however, it emerged as an important reason for not being willing to move in our survey and should therefore not be neglected. A second financial disincentive exists for tenants of cooperatives lacking clear occupancy rules (i.e. SCHL), which could be eliminated by imperatively linking cost rents to occupancy rules and rigorously enforcing them.

### Limitations

Several limitations to our conceptual approach, the survey analysis and the generalizability of the study should be noted.

Firstly, in our model of past moves, we assumed that observed changes in dwelling size were the result of a household’s *choice* of dwelling size. However, housing choice is ‘a choice under constraints’ and involves a trade-off between different dwelling characteristics (Rérat, 2020, pp. 225–226). As the choice between different options might have been very limited, aspects other than size might have been preponderant in the selection of the dwelling. Secondly, due to data availability, we have employed the risk approach, whereas other scholars modelled the residential mobility process with a two-stage approach (e.g. Clark & Lisowski, 2017; de Groot et al., 2011a, 2011b; Mulder, 1996). Although the risk approach is widely used, it does not enable discernment between intentions to move and actual moving behaviour. However, we enriched the study of actual moving behaviour in the first part of the study with an analysis of stated preferences in the second part. Furthermore, the advantage of the two-stage approach has been relativized through previous findings that the formation of the intention to move and the choice of a new dwelling may well take place simultaneously (e.g. Mulder, 1996). Thirdly, the expressed willingness to move due to a shrinking household was only hypothetical; therefore, the stated answers might differ from the behaviour tenants would show in a real-life situation, which is a common drawback of stated preference approaches. Lastly, this study is restricted to Swiss tenants of three different owners in mainly urban regions and did not aim for generalizability. Due to the substantial complexity of the formation of residential preferences and choices and their dependence on cultural, spatial and temporal contexts, opportunities and constraints for reducing housing size may vary between different contexts and between dwelling owners both within and outside of Switzerland.

### Future research: The environmental footprint of housing

As part of the overarching goal of this paper, we investigated the potential of reducing housing size as a means to increase housing sustainability by also considering its sociocultural dimension. Although research has confirmed that the per capita environmental impact of housing increases with growing space consumption, this relation is not linear. Depending on the type of dwelling, its energy standard and its construction materials, the relevance of dwelling size in its overall environmental impact varies. To further reduce the environmental footprint of housing, other aspects such as occupant behaviour, mobility, energy efficiency or the decarbonisation of heating systems should be taken into account (Dürrenberger et al., 2001; Guerra Santin et al., 2009; Perkins et al., 2009; Randolph, 2008; Saner et al., 2013). Furthermore, aside from the size of a dwelling unit, the number of occupants of the unit is also crucial. The per capita consumption of space and energy in housing usually decreases with increasing household size as space, infrastructure, goods and services are shared among more people (Dürrenberger et al., 2001; FSO, 2019a; Underwood & Zahran, 2015; c.f. Table 2). In consequence, future research should not only focus on studying how to reduce the size of individual dwelling units but also on possibilities for and social acceptance of more condensed building and living and sharing of resources among residents (as was also mentioned in Sect. 5.2.2). Doing so will be crucial to counteracting the increasing emergence of separate dwelling units as a consequence of the growing number of single and two-person households in Switzerland. In addition, future research should more deeply investigate the relationship between housing functions and the choice of dwelling size and use the resulting insights to develop dwelling concepts that fulfil diverse functions while also using space efficiently. Finally, to contribute to the global effort toward reducing housing’s environmental footprint, we invite researchers to explore the findings of this paper in different geographical and cultural contexts.

## Conclusion

In this study, we directed residential mobility research towards the broader question of how to reconcile environmentally sustainable housing with households’ needs and preferences. By means of a survey with tenants of two large cooperatives and of a private real estate owner in Switzerland, we investigated obstacles and opportunities for reducing housing size.

We revealed that a major obstacle for reducing dwelling size is the preference for large dwellings, particularly to fulfil the functions of ‘privacy’, ‘status symbol’ and ‘permanence’, in combination with the (financial) freedom to choose one’s dwelling. In addition, substantial non-monetary and monetary costs of moving can impede relocation. An opportunity for downsizing is seen in some tenants prioritizing the financial benefit of a lower rent as well as those who prefer smaller dwellings to fulfil the functions of ‘self-representation’ and ‘production-consumption’.

Accordingly, this paper underpins previous calls for invisible energy policies and offers sources of inspiration for such. More specifically, we argue for incentives for and facilitation of downsizing moves, as is currently practiced in cooperatives, in particular for tenants in the private rental market. An additional requirement is a sufficient supply of small dwellings that are centrally located and well-connected and fulfil diverse needs. For this purpose, denser building and living might be necessary, which would simultaneously increase the environmental sustainability of housing and allow more people to live in desired areas. Finally, we underline the need to rethink current housing standards to provide resource-efficient dwellings that also ensure a high quality of living and attract affluent households.

Future research should make use of the housing functions concept to elaborate housing forms that can meet diverse preferences while also using space efficiently, and investigate in how to counteract the environmental consequences of the growing number of single households with increased sharing of space and resources.

## Appendix

See Tables 6, 7, 8, 9, 10, 11, 12, 13 and 14.

**Table 6:** The nine housing functions and their definitions: after Pagani and Binder (2021)

Function label	Definition
Property	A place that belongs to the occupant, of which s/he is entitled to do what s/he wants
Production-consumption	A place that enables one to perform activities (like eating, laundering, companionship)
Impermanence	A place free from tradition or memory, which reflects one’s life stage
Status symbol	A credential for esteem, a place for exhibiting
Privacy	A private place mainly for the family's needs. The recreation preferably happens outside
Commodity	A temporary place or a starting point. Maybe attractive for its price or location
Self-representation	A place for self-expression, satisfaction of aspirations
Shelter	A refuge, a fortress where one can return to get rest, before going back out 'into the world'; the 'homely home'
Permanence	A place a person feels they belong or are rooted in

**Table 7:** Sample description: Household sociodemographic characteristics, affiliation with dwelling owners and space consumption per person

Variable [0,1]	Frequency	
	n	% or mean (S.D.)
*Gender*	877	100.0
Female	472	53.8
Mmale	405	46.2
*Age*	878	100.0
33 and younger	147	16.7
34–49	289	32.9
50–64	258	29.4
65 and older	184	21.0
*Marital status*	874	100.0
Ssingle	201	23.3
Mmarried/couple	438	50.1
dDivorced/separated/alone/widow	232	26.5
*Presence of children*	870	100.0
HH with children	239	27.5
HH without children	631	72.5
*Household size*	809	100.0
1 pers	269	33.3
2 pers	285	35.2
3–4 pers	216	26.7
5 + pers	39	4.8
*Annual income of household*	701	100.0
Below 60 K CHF	229	32.7
60 K – 88 K CHF	211	30.1
88 K – 120 K CHF	149	21.3
120 K – 165 K CHF	67	9.6
Above 165 K CHF	45	6.4
*Level of education*	811	100.0
Mandatory school not completed	4	0.5
Mandatory school	72	8.9
Professional/commercial school	319	39.3
High school (Matura)	53	6.5
University (Bachelor/Master)	326	40.2
PhD	37	4.6
*Dwelling owner*	878	100.0
ABZ	294	33.5
SCHL	347	39.5
SM	237	27.0
*Space consumption [m* ^*2*^ */cap]*	875	45.8 (20.9)

**Table 8:** Change in household size after the previous move in relation to the trigger inducing the move

	Change in HH size				
	n	% reduced	% augmented	% no change	Sign
Full sample By trigger	862	39.1	15.9	45.0	
Raise in salary	10	30.0	0.0	70.0	
Retirement	10	20.0	10.0	70.0	
Opportunity to rent	104	32.7	10.6	56.7	**
Accessibility	13	15.4	0.0	84.6	**
New job location	49	30.6	20.4	49.0	
Rental contract expiration	17	47.1	5.9	47.1	
Interpersonal problems	12	33.3	8.3	58.3	
Increasing lack of comfort	100	31.0	10.0	59.0	***
Need for radical change in life	22	22.7	27.3	50.0	
Rent too high	54	38.9	9.3	51.9	
Forced move	64	31.3	6.3	62.5	***
Lack of space	50	24.0	16.0	60.0	*
Family (ageing, children)	10	30.0	0.0	70.0	
Divorce, separation, loss of partner	70	91.4	1.4	7.1	***
Moving in with partner	89	29.2	38.2	32.6	***
New child	95	21.1	43.2	35.8	***
Children leaving home	44	77.3	2.3	20.5	***
Leaving parents' home	9	100.0	0.0	0.0	***
Need for autonomy	27	77.8	3.7	18.5	***

***, **, * indicate the 1%, 5% and 10% significance levels, respectively;

HH = household

**Table 9:** Change in dwelling size in relation to change in housing functions during the last move. Only cases in which the household size did not change are considered

By change in housing functions	n	%	Change in m^2^		
	Total	388	Increase (%)	Decrease (%)	Sign
			69.8	30.2	
Property					
+	74	19.1	78.4	21.6	
-	54	13.9	55.6	44.4	**
=	260	67.0	70.4	29.6	
Production-consumption					
+	107	27.6	78.5	21.5	**
-	33	8.5	57.6	42.4	
=	248	63.9	67.7	32.3	
Impermanence					
+	90	23.2	71.1	28.9	
-	112	28.9	72.3	27.7	
=	186	47.9	67.7	32.3	
Status symbol					
+	78	20.1	84.6	15.4	***
-	65	16.8	60.0	40.0	*
=	245	63.1	67.8	32.2	
Privacy					
+	101	26.0	78.2	21.8	**
−	51	13.1	68.6	31.4	
=	236	60.8	66.5	33.5	*
Commodity					
+	70	18.0	67.1	32.9	
-	140	36.1	75.0	25.0	
=	178	45.9	66.9	33.1	
Self-representation					
+	140	36.1	78.6	21.4	***
-	67	17.3	68.7	31.3	
=	181	46.6	63.5	36.5	**
Shelter					
+	134	34.5	75.4	24.6	
-	39	10.1	61.5	38.5	
=	215	55.4	67.9	32.1	
Permanence					
+	149	38.4	73.8	26.2	
-	58	14.9	62.1	37.9	
=	181	46.6	69.1	30.9	

**, ** and ** indicate the 1%, 5% and 10% significance levels

**Table 10:** Change in dwelling size in relation to the trigger inducing the move

	Change in m^2^				
	n	%	% reduced	% augmented	Sign
Full sample By trigger	864	100.0	40.0	60.0	
Raise in salary	10	1.2	10.0	90.0	
Retirement	10	1.2	70.0	30.0	
Opportunity to rent	104	12.0	22.1	77.9	***
Accessibility	13	1.5	23.1	76.9	
New job location	49	5.7	53.1	46.9	*
Rental contract expiration	17	2.0	52.9	47.1	
Interpersonal problems	12	1.4	33.3	66.7	
Increasing lack of comfort	100	11.6	31.0	69.0	*
Need for radical change in life	22	2.5	45.5	54.5	
Rent too high	55	6.4	58.2	41.8	***
Forced move	65	7.5	38.5	61.5	
Lack of space	50	5.8	8.0	92.0	***
Family (ageing, children)	10	1.2	20.0	80.0	
Divorce, separation, loss of partner	70	8.1	84.3	15.7	***
Moving in with partner	89	10.3	34.8	65.2	
New child	95	11.0	6.3	93.7	***
Children leaving home	44	5.1	86.4	13.6	***
Leaving parents' home	9	1.0	100.0	0.0	***
Need for autonomy	27	3.1	74.1	25.9	***

***, **, * indicate the 1%, 5% and 10% significance levels, respectively

**Table 11:** Binary logistic regression models with the dependent variable ‘household reduced dwelling size upon the last relocation’

Variable	Model 1			Model 2			Model 3		
	B	S.E	exp(B)	B	S.E	exp(B)	B	S.E	exp(B)
CHANGE IN HOUSEHOLD SIZE (ref. cat. no change)									
Increase	− 0.712***	0.257	0.491***	− 0.461	0.281	0.631	− 0.445	0.286	0.641
decrease	1.245***	0.162	3.473***	0.829***	0.183	2.291***	0.822***	0.187	2.275***
CHANGE IN HOUSING FUNCTIONS									
Impermanence +	0.385**	0.178	1.470**				0.202	0.197	1.224
status symbol +	− 0.486**	0.211	0.615**				− 0.473**	0.227	0.623**
Status symbol -	0.587***	0.205	1.799***				0.615***	0.227	1.849***
Privacy +	− 0.484***	0.181	0.616***				− 0.418**	0.200	0.658**
Shelter -	0.465*	0.242	1.593*				0.26	0.263	1.297
TRIGGERS									
Opportunity to rent				− 1.022***	0.315	0.360***	− 0.929***	0.323	0.395***
New job location				0.487	0.359	1.627	0.537	0.367	1.711
Increasing lack of comfort		− 0.532*	0.3	0.588*	− 0.461	0.308	0.631		
Rent too high				0.629*	0.349	1.875*	0.634*	0.359	1.886*
Lack of space				− 2.135***	0.564	0.118***	− 2.100***	0.568	0.122***
Forced move				− 0.247	0.333	0.781	− 0.335	0.345	0.715
Divorce, separation, loss of partner		1.465***	0.398	4.329***	1.453***	0.406	4.274***		
Moving in with partner		− 0.217	0.314	0.805	− 0.267	0.321	0.765		
New child				− 2.270***	0.475	0.103***	− 2.260***	0.483	0.104***
Children leaving home		1.782***	0.491	5.943***	1.742***	0.498	5.706***		
Need for autonomy				0.968*	0.495	2.632*	0.980*	0.509	2.666*
Constant	− 0.893***	0.139	0.410***	− 0.513**	0.217	0.599**	− 0.528**	0.246	0.590**
n	849			849			849		
df	7			13			18		
Chi2	138.324***		258.267***		282.020***				
−LL2	1004			884.02			860.27		
Nagelkerke pseudo R2	0.203			0.355			0.382		
AIC	1020			912.02			898.27		

***, ** and * indicate the 1%, 5% and 10% significance levels

**Table 12:** Descriptive statistics of categories of willingness to move and bivariate relations between categories of willingness to move and independent variables

	n	% *or* mean (S.D.) in sample			% *or* mean (S.D.) in subsample	sign
*Willingness to move*	570	100.0	not willing	neutral	willing	
Not willing	220	38.6				
Neutral	207	36.3				
Willing	143	25.1				
CURRENT HOUSING FUNCTIONS	570					
Property		2.66 (1.18)	2.64 (1.19)	2.73 (1.179)	2.57 (1.17)	
Production-consumption		4.31 (0.68)	4.27 (0.70)	4.25 (0.69)	4.45 (0.61)	** (n-w, nw-w)
Impermanence		3.02 (1.10)	3.04 (1.11)	3.02 (1.03)	2.99 (1.20)	
status symbol		2.06 (0.99)	2.13 (0.96)	2.14 (1.08)	1.84 (0.85)	** (nw-w)
Privacy		3.67 (0.88)	3.67 (0.88)	3.70 (0.87)	3.65 (1.00)	
Commodity		3.08 (1.20)	3.10 (1.23)	3.09 (1.12)	3.06 (1.25)	
Self-representation		3.32 (0.99)	3.28 (1.01)	3.33 (1.00)	3.35 (1.02)	
Shelter		3.93 (0.92)	3.98 (0.87)	3.87 (0.95)	3.94 (0.93)	
Permanence		3.47 (1.08)	3.57 (1.08)	3.43 (1.07)	3.37 (1.09)	
MICRO CONTEXT (household)						
*Gender*	570	100.0				
Female	294	51.6	55.0	45.4	55.2	*
Male	276	48.4	45.0	54.6	44.8	*
*Age*	570	100.0				
33 and younger	94	16.5	17.3	15.9	16.1	
34–49	213	37.4	41.4	31.4	39.9	*
50–64	176	30.9	22.7	36.7	35.0	***
65 and older	87	15.3	18.6	15.9	9.1	**
*Marital status*	567	100.0				
Single	62	10.9	12.4	9.2	11.3	
Married or couple	408	72.0	69.7	74.4	71.8	
Divorced, separated, alone or widowed	97	17.1	17.9	16.4	16.9	
*Presence of children*	565	100.0				
HH with children	237	41.9	41.7	36.4	50.3	**
HH without children	328	58.1	58.3	63.6	49.7	**
* No. of persons in HH*	567	100.0				
1^a^	0	0.0				
2	282	49.7	53.2	50.5	43.4	
3–4	241	42.5	40.4	41.3	47.6	
5 +	42	7.4	6.4	7.3	9.1	
* Annual income of household*	448	100.0				
Below 60 K CHF	97	21.7	20.3	23.5	21.2	
60 K – 88 K CHF	141	31.5	26.6	34.0	35.6	
88 K – 120 K CHF	114	25.4	24.3	25.5	27.1	
120 K – 165 K CHF	57	12.7	16.4	9.2	11.9	
Above 165 K CHF	39	8.7	12.4	7.8	4.2	**
*Prospect of moving within the next 5 years*	551	100.0				
Yes/maybe	236	42.8	42.3	38.1	50.4	*
No	315	57.2	57.7	61.9	49.6	*
*Willingness to move*	570	100.0	Not willing	Neutral	Willing	
Not willing	220	38.6				
Neutral	207	36.3				
Willing	143	25.1				
MICRO CONTEXT (dwelling)						
*Area of dwelling*	570	90 (20)	88 (19)	91 (18)	94 (23)	*** (nw-w)
*m* ^*2*^ * per person*	567	35 (10)	34 (10)	35 (10)	34 (11)	
*Level of satisfaction*	570	100.0				
Unsatisfied	81	14.2	12.3	16.4	14.0	
Neutral	29	5.1	5.5	6.3	2.8	
Satisfied	460	80.7	82.3	77.3	83.2	
MACRO CONTEXT (market)						
*Owner*	570	100.0				
ABZ	213	37.4	32.3	35.3	48.3	***
SCHL	202	35.4	42.3	32.9	28.7	**
SM	155	27.2	25.5	31.9	23.1	

^a^The willingness to move in case of a shrinking household was only assessed for those households with more than one person; ***, ** and * indicate the 1%, 5% and 10% significance levels, respectively; categories of willingness to move between which the independent variable differed significantly, are indicated in brackets; nw = not willing; n = neutral; w = willing

**Table 13:** Multinomial logistic regression models with willingness to move in case the household size decreased as the dependent variable and the reference category ‘not willing to move’. The regression parameters, standard errors and odds ratios of four different models are shown; for each model, a set of parametssers for the regression between the reference category and the other respective category is given. The bottom section shows the number of observations included in the analysis (*n*), the model statistics and measures of model fit

Variable	Model 1		Model 2		Model 3		Model 4	
	Neutral	Willing	Neutral	Willing	Neutral	willing	Neutral	Willing
***CURRENT HOUSING FUNCTIONS***								
Property	0.212* (0.111) [1.236*]	0.114 (0.117) [1.121]	0.212* (0.118) [1.236*]	0.065 (0.124) [1.068]	0.195 (0.12) [1.215]	0.065 (0.127) [1.067]	0.158 (0.123) [1.172]	− 0.024 (0.133) [0.976]
Production-consumption	− 0.022 (0.177) [0.978]	0.526** (0.213) [1.691**]	0.023 (0.186) [1.023]	0.632*** (0.221) [1.882***]	0.007 (0.189) [1.007]	0.603*** (0.224) [1.827***]	− 0.037 (0.193) [0.963]	0.620*** (0.23) [1.858***]
Impermanence	0.113 (0.111) [1.12]	0.014 (0.118) [1.014]	0.117 (0.115) [1.125]	0.043 (0.122) [1.044]	0.124 (0.117) [1.133]	0.039 (0.125) [1.04]	0.116 (0.118) [1.123]	0.035 (0.127) [1.036]
Status symbol	0.021 (0.122) [1.021]	− 0.394*** (0.143) [0.675***]	0.099 (0.129) [1.105]	− 0.352** (0.147) [0.704**]	0.108 (0.131) [1.114]	− 0.360** (0.149) [0.698**]	0.105 (0.133) [1.111]	− 0.343** (0.153) [0.710**]
Privacy	0.101 (0.135) [1.106]	− 0.061 (0.138) [0.94]	0.104 (0.139) [1.109]	− 0.093 (0.144) [0.911]	0.138 y(0.139) [1.148]	− 0.065 (0.148) [0.937]	0.197 (0.143) [1.217]	0.007 (0.152) [1.007]
Commodity	− 0.05 (0.105) [0.952]	− 0.078 (0.112) [0.925]	− 0.042 (0.11) [0.959]	− 0.136 (0.118) [0.873]	− 0.043 (0.112) [0.958]	− 0.106 y(0.121) [0.899]	− 0.056 (0.115) [0.945]	− 0.171 (0.125) [0.843]
Self-representation	0.157 (0.138) [1.17]	0.309** (0.149) [1.362**]	0.112 (0.147) [1.118]	0.355** (0.158) [1.426**]	0.106 (0.149) [1.111]	0.325** (0.161) [1.383**]	0.138 (0.15) [1.148]	0.376** (0.164) [1.456**]
Shelter	− 0.264* (0.147) [0.768*]	− 0.007 (0.161) [0.993]	− 0.217 (0.156) [0.805]	0.02 (0.168) [1.02]	− 0.256 (0.16) [0.774]	− 0.04 (0.172) [0.961]	− 0.270* (0.16) [0.764*]	− 0.122 (0.174) [0.886]
Permanence	− 0.136 (0.133) [0.873]	− 0.385*** (0.142) [0.680***]	− 0sss.217 (0.141) [0.805]	− 0.441*** (0.153) [0.643***]	− 0.193 (0.144) [0.825]	− 0.460*** (0.156) [0.632***]	− 0.231 (0.151) [0.794]	− 0.589*** (0.165) [0.555***]
***MICRO CONTEXT (household)***								
*Age (ref. cat. 34–49)*								
33 and younger			0.044 (0.391) [1.045]	− 0.466 (0.417) [0.627]	0.191 (0.398) [1.21]	− 0.361 (0.429) [0.697]	0.238 (0.403) [1.269]	− 0.191 (0.445) [0.826]
50–64			0.787** (0.319) [2.197**]	0.334 (0.34) [1.396]	0.794** (0.322) [2.213**]	0.287 (0.347) [1.332]	0.806** (0.323) [2.238**]	0.286 (0.353) [1.331]
65 and older			0.139 (0.419) [1.149]	− 0.566 (0.494) [0.568]	0.211 (0.424) [1.235]	− 0.495 (0.503) [0.609]	0.265 (0.427) [1.304]	− 0.429 (0.511) [0.651]
*HH with children (ref. cat. without children*								
	− 0.403 (0.285) [0.669]	0.052 (0.308) [1.053]	− 0.613** (0.299) [0.542**]	− 0.155 (0.324) [0.856]	− 0.593** (0.301) [0.553**]	− 0.189 (0.332) [0.828]		

Variable	Model 1		Model 2		Model 3		Model 4	
	Neutral	Willing	Neutral	Willing	Neutral	Willing	Neutral	Willing
*Marital status (ref. cat. married or couple)*								
Single			0.024 (0.441) [1.024]	0.022 (0.455) [1.023]	0.182 (0.451) [1.199]	0.252 (0.468) [1.287]	0.091 (0.453) [1.095]	0.2 (0.482) [1.221]
Divorced separated alone or widowed		0.083 (0.347) [1.086]	− 0.435 (0.39) [0.647]	0.091 (0.35) [1.095]	− 0.407 (0.395) [0.666]	0.098 (0.352) [1.103]	− 0.412 (0.4) [0.662]	
*Annual income of household (ref. cat. 60 K—88 K CHF)*								
Below 60 K CHF			− 0.075 (0.35) [0.928]	− 0.076 (0.385) [0.927]	0.098 (0.361) [1.103]	0.093 (0.396) [1.098]	0.097 (0.362) [1.101]	0.071 (0.405) [1.073]
88 K–120 K CHF			− 0.174 (0.323) [0.84]	− 0.416 (0.346) [0.66]	− 0.272 (0.337) [0.762]	− 0.659* (0.362) [0.518*]	− 0.293 (0.34) [0.746]	− 0.628* (0.373) [0.534*]
120 K – 165 K CHF			− 0.782* (0.414) [0.457*]	− 0.836** (0.423) [0.433**]	− 1.029** (0.433) [0.357**]	− 1.244*** (0.449) [0.288***]	− 1.075** (0.441) [0.341**]	− 1.107** (0.462) [0.331**]
Above 165 K CHF			− 0.656 (0.452) [0.519]	− 1.914*** (0.591) [0.147***]	− 1.037** (0.486) [0.355**]	− 2.427*** (0.631) [0.088***]	− 1.085** (0.496) [0.338**]	− 2.354*** (0.646) [0.095***]
*Prospect of moving within the next 5 years*			− 0.185 (0.276) [0.831]	0.524* (0.296) [1.688*]	− 0.109 (0.284) [0.897]	0.700** (0.305) [2.014**]	− 0.11 (0.286) [0.896]	0.726** (0.31) [2.068**]
***MICRO CONTEXT (dwelling)***								
*Area of dwelling [m* ^*2*^ *]*					0.020*** (0.008) [1.020***]	0.025*** (0.008) [1.025***]	0.018** (0.008) [1.019**]	0.024*** (0.008) [1.025***]
*Level of satisfaction (ref. cat. satisfied)*								
Unsatisfied					0.382 (0.351) [1.465]	0.021 (0.396) [1.021]	0.253 (0.358) [1.288]	− 0.194 (0.409) [0.824]
neutral					0.768 (0.574) [2.156]	− 1.193 (0.873) [0.303]	0.778 (0.57) [2.176]	− 1.07 (0.875) [0.343]
***MACRO CONTEXT (market)***								
*Owner (ref. cat. ABZ)*								
SCHL							− 0.565* (0.315) [0.569*]	− 1.218*** (0.346) [0.296***]
SM							− 0.034 (0.36) [0.967]	− 0.883** (0.394) [0.414**]
*Intercept*	− 0.18 (0.926) [0.8350]	− 1.412 (1.103) [0.2440]	− 0.139 (1.004) [0.870]	− 1.446 (1.186) [0.2360]	− 1.893 (1.193) [0.151]	− 3.214** (1.373) [0.040**]	− 1.316 (1.246) [0.268]	− 1.844 (1.436) [0.158]
*n*	430		430		430		430	
df	18		40		46		50	
Chi^2^	40.746***		82.444***		102.048***		117.545	
−2LL	891.646		852.721		833.117		817.62	
**Nagelkerke pseudo R** ^**2**^	**0.102**		**0.197**		**0.238**		**0.270**	
**AIC**	**931.646**		**936.721**		**929.117**		**921.62**	

**Table 14:** Relations between income categories and household characteristics and the dwelling owner

			Below 60 K CHF	60 K – 88 K CHF	88 K – 120 K CHF	120 K – 165 K CHF	above 165 K CHF	Frequency in sample (%)
	Frequency in sample (%)		21.7	31.5	25.4	12.7	8.7	
Frequency in income category (%)	*Age*		(n = 448)					
		33 and younger	13.4	13.5	21.9	24.6	20.5	17.6
		34–49	34	34.8	35.1	38.6	59	37.3
		50–64	33	26.2	31.6	33.3	20.5	29.5
		65 and older	19.6	25.5	11.4	3.5	0.0	15.6
	Sign			***		**	***	
	*Marital status*		(n = 445)					
		Single	14.7	8.5	13.3	10.5	12.8	11.7
		Married or couple	48.4	77.3	72.6	82.5	84.6	71.2
		Divorced, separated, alone or widowed	36.8	14.2	14.2	7.0	2.6	17.1
	Sign		***			*	**	
	*Presence of children*		(n = 443)					
		HH with children	38.3	39.3	51.3	50.9	53.8	44.9
		HH without children	61.7	60.7	48.7	49.1	46.2	55.1
	sign							
	*Owner*		(n = 448)					
		ABZ	50.5	43.3	30.7	22.8	23.1	37.3
		SCHL	37.1	39	35.1	31.6	30.8	35.9
		SM	12.4	17.7	34.2	45.6	46.2	26.8
	sign		***	**	*	***	**	

***, ** and * indicate the 1%, 5% and 10% significance levels, respectively;

HH = household

## Footnotes

1 For instance, the framework does not depict the concept of residential satisfaction, which influences and is influenced by both triggers and housing functions (Pagani et al., 2021).

2 Two of the largest housing cooperatives in Switzerland are the *Allgemeine Baugenossenschaft Zürich* (ABZ; https://www.abz.ch/genossenschaft/portrait/; accessed 27.10.20) and the *Société Coopérative d’Habitation Lausanne* (SCHL; https://www.schl.ch/; accessed 27.10.20), which are partners in this research.

3 This is practiced in ABZ.

4 We did not consider education and employment status in the regression because these applied only to the person completing the survey, whereas the relocation decision concerned the entire household.

5 The test of parallel lines, or proportional odds assumption, verifies whether the regression parameters are the same between all categories of the dependent variable. For more details, see https://www.ibm.com/support/knowledgecenter/en/SSLVMB_23.0.0/spss/tutorials/plum_germcr_parallel.html#plum_germcr_parallel (accessed 16.12.2020).

6 However, it must be noted that the dwelling area as well as the housing functions are interval variables and that their effects increase with larger variable variation.
